# A concise guide to developing and using quantitative models in conservation management

**DOI:** 10.1002/csp2.11

**Published:** 2019-04-15

**Authors:** Pablo García-Díaz, Thomas A.A. Prowse, Dean P. Anderson, Miguel Lurgi, Rachelle N. Binny, Phillip Cassey

**Affiliations:** 1 Manaaki Whenua - Landcare Research, Lincoln, New Zealand; 2 School of Mathematical Sciences, The University of Adelaide, North Terrace, South Australia, Australia; 3 Centre for Biodiversity Theory and Modelling, Theoretical and Experimental Ecology Station, CNRS-Paul Sabatier University, Moulis, France; 4 Te Pūnaha Matatini, Centre of Research Excellence for Complex Systems and Networks, Auckland, New Zealand; 5 School of Biological Sciences, The University of Adelaide, North Terrace, South Australia, Australia

**Keywords:** applied conservation, ecological models, prediction, projection, simulation model, statistical model, uncertainty

## Abstract

Quantitative models are powerful tools for informing conservation management and decision-making. As applied modeling is increasingly used to address conservation problems, guidelines are required to clarify the scope of modeling applications and to facilitate the impact and acceptance of models by practitioners. We identify three key roles for quantitative models in conservation management: (a) to assess the extent of a conservation problem; (b) to provide insights into the dynamics of complex social and ecological systems; and, (c) to evaluate the efficacy of proposed conservation interventions. We describe 10 recommendations to facilitate the acceptance of quantitative models in conservation management, providing a basis for good practice to guide their development and evaluation in conservation applications. We structure these recommendations within four established phases of model construction, enabling their integration within existing workflows: (a) design (two recommendations); (b) specification (two); (c) evaluation (one); and (d) inference (five). Quantitative modeling can support effective conservation management provided that both managers and modelers understand and agree on the place for models in conservation. Our concise review and recommendations will assist conservation managers and modelers to collaborate in the development of quantitative models that are fit-for-purpose, and to trust and use these models appropriately while understanding key drivers of uncertainty.

## 1 Introduction

Implementing effective *conservation management* is crucial in the face of the current biodiversity crisis (Ceballos, Ehrlich, & Dirzo, 2017; Groves & Game, 2016; Pimm et al., 2014; Waldron et al., 2013). Expert opinion, drawn from the experience of conservation managers, is commonly used to develop and implement conservation actions, yet research has shown that the outcomes of these actions can be improved if complemented with *quantitative models* (Addison et al., 2013; Cook, Hockings, & Carter, 2009; Martin et al., 2012; Pullin, Knight, Stone, & Charman, 2004; Rose et al., 2018; Sutherland & Wordley, 2017). Indeed, quantitative models can produce better conservation management than expertise-based actions (Addison et al., 2013; Holden & Ellner, 2016; McCarthy et al., 2004). A critical role for quantitative modeling in applied conservation has been recently highlighted by the Intergovernmental Science-Policy Platform on Biodiversity and Ecosystem Services (IPBES)(Akçakaya et al., 2016) and is analogous to the indispensable role of climate modeling for the assessments made by the Intergovernmental Panel on Climate Change (Pachauri et al., 2014).

The increased availability of open-access modeling softwares, such as packages built for the R statistical and graphical computing environment (Kéry & Royle, 2016; R Development Core Team, 2015) and the Maxent *species distribution modeling* software (Phillips, Anderson, & Schapire, 2006; Yackulic et al., 2013), have fostered the widespread application of quantitative models in ecology and conservation. Modeling tools are being used by specialists but also by non-modelers and conservation managers with little quantitative training (Barraquand et al., 2014; Conroy & Peterson, 2013; Dietze et al., 2018; Schmolke, Thorbek, DeAngelis, & Grimm, 2010; Touchon & McCoy, 2016; Yackulic et al., 2013). As a result, quantitative models are rapidly becoming entrenched in the toolbox of conservation practice, policy, and management (Akçakaya et al., 2016; Conroy & Peterson, 2013; Getz et al., 2018; Guisan et al., 2013; Law et al., 2017; Nicholson et al., 2018; Schmolke et al., 2010). In fact, quantitative models are fundamental components of some formal conservation decision-making frameworks (Conroy & Peterson, 2013; Schwartz et al., 2018). For example, quantitative models are critical to structured decision-making, where they are used to *predict* how natural systems will respond to conservation actions and to optimize such actions to achieve conservation goals (Addison et al., 2013; Conroy & Peterson, 2013; McCarthy et al., 2010; Wilson, Carwardine, & Possingham, 2009). Furthermore, meta-analyses are a natural way to synthesise evidence for the effectiveness of conservation actions; a fundamental component of the evidence-based conservation paradigm (Cassey, Delean, Lockwood, Sadowski, & Blackburn, 2018; Pullin & Stewart, 2006; Schwartz et al., 2018).

Unfortunately, the increased popularity of quantitative modeling has not always resulted in better conservation outcomes. Poor modeling practices can result in inappropriate inferences and serious unintended, potentially detrimental, consequences for conservation management (Addison et al., 2013; Barraquand et al., 2014; Bestelmeyer, 2006; Coulson, Mace, Hudson, & Possingham, 2001; Harihar, Chanchani, Pariwakam, Noon, & Goodrich, 2017; Moilanen, 2011; Sofaer, Jarnevich, & Flather, 2018; Touchon & McCoy, 2016; Wilson, Westphal, Possingham, & Elith, 2005). Therefore, it is important to keep in mind the often-cited premise expressed by George E. P. Box (Box, 1979): “All models are wrong, but some are useful”. The effective uptake and application of quantitative models in conservation management requires both sound modeling practices and substantial trust from conservation practitioners that such models are reliable and valuable tools for informing their time- and cost-critical tasks (Addison et al., 2013; Conroy & Peterson, 2013; Dietze, 2017; Getz et al., 2018; Holden & Ellner, 2016; Nicholson et al., 2018; Parrott, 2017; Schmolke et al., 2010). In particular, greater understanding of the many ways in which quantitative models can improve on-ground conservation actions will facilitate their acceptance by managers. In this fashion, we aim to provide a general overview of the application of quantitative models in conservation management and introduce a series of recommendations for improving the integration of quantitative modeling within conservation practice. Our concise review will be especially useful for researchers and conservation managers who are beginning to use quantitative models or who use them infrequently, although we also expect our recommendations to be helpful and relevant for more experienced modelers. The references cited throughout the manuscript can be resourced to seek additional details and expand on the topics mentioned here. Importantly, we also envisage that our approach will facilitate greater communication between managers and modelers and to inform the effective adoption of best practice conservation decision-making.

We suggest using our review alongside those previously published by Schmolke et al. (2010), Dormann et al. (2012), Addison et al. (2013), and Law et al. (2017), whose perspectives and recommendations provide complementary and additional details on specific methods, for example, on species distribution models (Dormann et al., 2012). In the context of environmental decision-making, Schmolke et al. (2010) and Addison et al. (2013) conducted reviews of quantitative modeling and listed their own recommendations. It is not a surprise that some of our recommendations overlap with those previously published (see Table 1), but we also provide our own unique recommendations and a framework for guiding them.

## 2 A Concise Taxonomy of Quantitative Models

**Figure 1:**
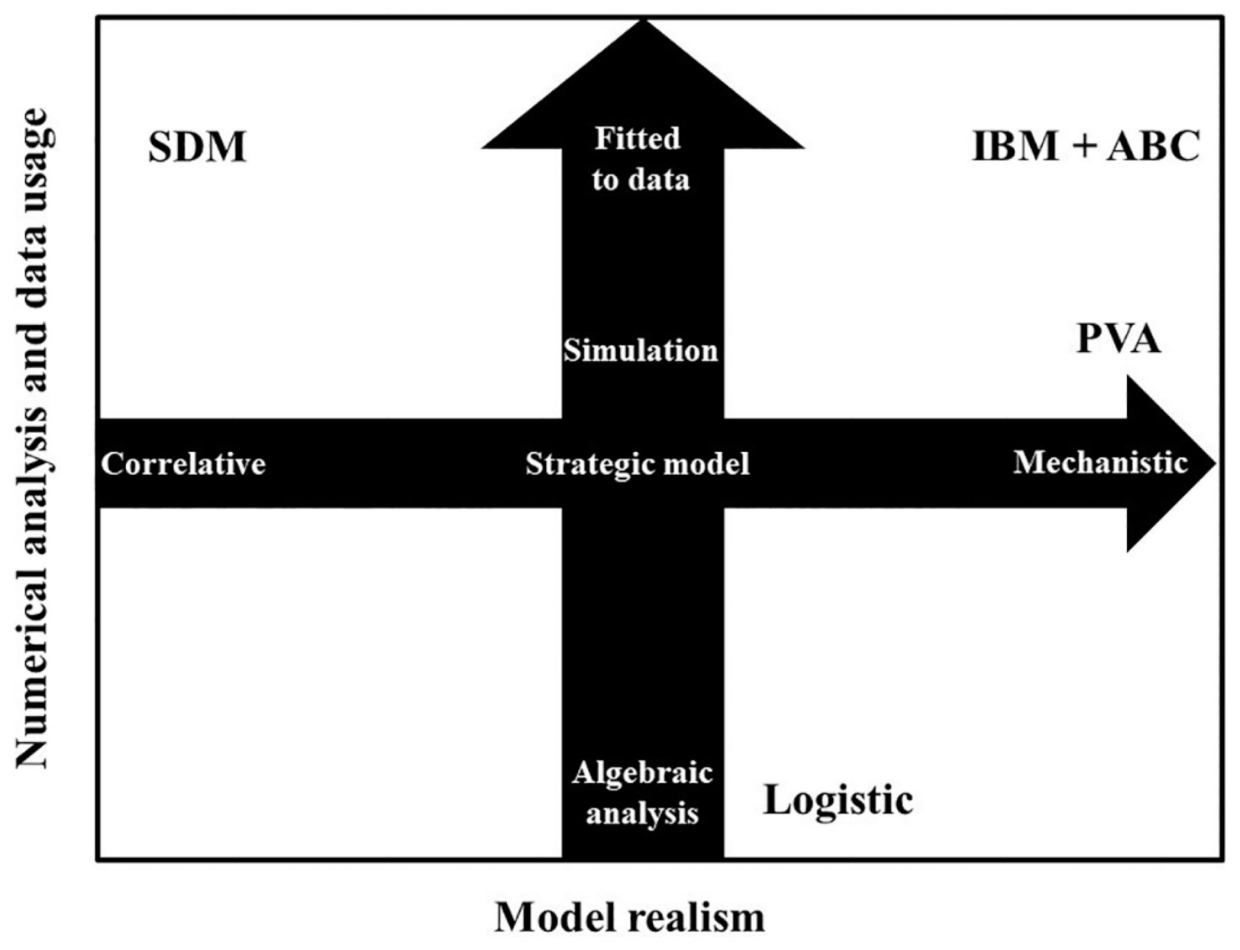
A classification of quantitative models based on their realism (increasing from left to right) and the analytical approach taken to investigate them (increasing from bottom to top). Acronyms indicate the approximate position of some exemplary models (see also main text): SDM: a correlative species distribution model; IBM + ABC: spatially-explicit individual-based model fitted to data using approximate Bayesian computation procedures; PVA: population viability analysis using the VORTEX software; and, logistic: algebraic analysis of a logistic population growth model

Quantitative modeling encompasses a broad array of approaches, and there have been many classifications and a large array of associated terms used to describe quantitative models (Dormann et al., 2012; Evans et al., 2013; Fordham et al., 2018; Getz et al., 2018; Hobbs & Hooten, 2015; Wood, 2001). Nevertheless, most classifications of quantitative models typically combine features of two main axes, which are sufficient to recognize differences between quantitative models and establish the basis for the simplified taxonomy that frames this review (Figure 1). The first axis quantifies the level of realism or model detail, which is largely determined by the specification and description of the mechanisms producing the processes and the patterns being modeled. Highly detailed *mechanistic models* include *individual-based models*, such as those exploring potential strategies for using *gene-drives* to eradicate populations of invasive species, whereas *correlative models* are examples of more simple models (DeAngelis & Yurek, 2017; Dormann et al., 2012; Evans et al., 2013; Peck, 2000; Prowse et al., 2017). *Strategic models* (sensu Evans et al., 2013), such as the well-known logistic population growth model, lie between the two extremes (Brook et al., 2000; Evans et al., 2013; Turchin, 2003).

The second axis describes the extent of numerical analysis and data usage in the modeling approach, including how the *parameters* are assigned values (sometimes also termed “calibration”, “model fitting”, “populating the model”, and “parameterization”; see Figure 1 and Glossary) (Dormann et al., 2012; Evans et al., 2013; Hobbs & Hooten, 2015; Wood, 2001). At one extreme, there are models calibrated or fitted to existing data using a variety of analytical and statistical methods (*statistical models* for simplicity, hereafter), for example, maximum likelihood estimation. General linear models (e.g., logistic regression) and generalized linear models (e.g., Poisson-log regression) for modeling species distributions and abundances are a notable example of statistical models widely used in conservation research (Dormann et al., 2012; Guisan et al., 2013; Kéry & Royle, 2016; Loiselle et al., 2003; Lurgi, Brook, Saltre, & Fordham, 2015; Renner et al., 2015; Sofaer et al., 2018; Tulloch et al., 2016; Warton et al., 2015; Wilson et al., 2005). At the opposite side of the spectrum on this second scale, we find *simulation models*. *Population viability analyses* conducted using, for example, the VORTEX individual-based software are well-known examples of applied simulation models (Beissinger & McCullogh, 2002; Brook et al., 2000; Lacy, 1993; Lurgi et al., 2015; McCarthy, Andelman, & Possingham, 2003). While highly flexible, such simulation models are often intractable mathematically. Some quantitative models are more readily amenable to purely theoretical analyses (e.g., using algebraic manipulations), which are not dependent on empirical data, for example in *differential equations* to identify threshold parameter values where the model behavior changes or to explore long-term behavior (Mangel, 2006).

## 3 Quantitative Models in Conservation Management

First and foremost, it is fundamental to understand the roles that quantitative models can play in conservation management, and the main features that determine their success in such roles. In general, quantitative models can fulfill two purposes in conservation management and policy; namely to diagnose the magnitude of a conservation issue and to assess the effectiveness of ongoing or future interventions (Cairney, 2016; Conroy & Peterson, 2013; Nicholson et al., 2018). More specifically, effective conservation modeling has the potential to:

1. Provide fundamental insights into the dynamics of both target species and ecological systems (Conroy & Peterson, 2013; Evans et al., 2013; Salafsky, Margoluis, & Redford, 2016; Saunders, Cuthbert, & Zipkin, 2018);
2. Help account for the complexities of real-world conservation management, characteristic of “*wicked*” *conservation problems* (Evans et al., 2013; Groves & Game, 2016; Parrott, 2017; Woodford et al., 2016); and
3. Offer a transparent, systematic, and repeatable way to assess, contrast and project the potential efficacy of conservation management solutions (Holden & Ellner, 2016; Law et al., 2017; McCarthy et al., 2004).

Quantitative models can support the achievement of these goals by both *estimating* parameters of interest, and predicting the dynamics of the target system under a variety of different conditions and “real-world” scenarios. There are abundant examples of quantitative models used in conservation management, but here we provide three examples to showcase the scope of their application:

1. Fisheries management routinely employs quantitative models to guide sustainable harvesting quotas (Walters & Maguire, 1996; Pauly et al., 2002; Costello, Gaines, & Lynham, 2008; Bradshaw et al., 2018; see also the publications of the International Commission for the Conservation of Atlantic Tunas: https://www.iccat.int/en/assess.htm). Incidentally, quantitative fisheries stock assessment also provides a real-life example of the dangers of potentially inadequate models. As highlighted by Addison et al. (2013), overly optimistic model-based estimates of Atlantic cod (*Gadus morhua*) abundance resulted in the over-exploitation of its Canadian stock (Walters & Maguire, 1996).
2. The global trade in plants and wildlife poses a severe risk to importing jurisdictions (García-Díaz & Cassey, 2017), because these species can become invasive or vector diseases (García-Díaz, Ross, Woolnough, & Cassey, 2017; Hulme, 2009, 2014; Jones et al., 2008; Martel et al., 2014). To reduce these risks, authorities around the world have instituted risk assessments to allow or ban the import of species based on quantitative or semi-quantitative models predicting the likelihood that the species will establish self-sustaining wild populations and/or produce severe impacts (Blackburn et al., 2014; Kumschick & Richardson, 2013; Lodge et al., 2016). Australia, the United States, and the European Union are amongst the jurisdictions using this methodology to risk management (Bomford, 2008; Hulme, Pyšek, Nentwig, & Vilà, 2009; Lodge et al., 2016).
3. In New Zealand, efforts deployed by the government Department of Conservation to control invasive mammal populations during their “Battle for our Birds” campaign are directly informed by an ecological model (Elliott & Kemp, 2016). Southern beech (*Nothofagus spp.*) mega-mast seeding in New Zealand produce an abundance of resources, which increases invasive small mammal consumer densities (Elliott & Kemp, 2016). The likelihood of a masting event is forecasted using a quantitative model, and control efforts are increased during the years with high predicted likelihood of a masting event (Elliott & Kemp, 2016; Kelly et al., 2013).

## 4 Toward Ensuring Best Practice in Quantitative Modeling for Conservation Management

**Figure 2:**
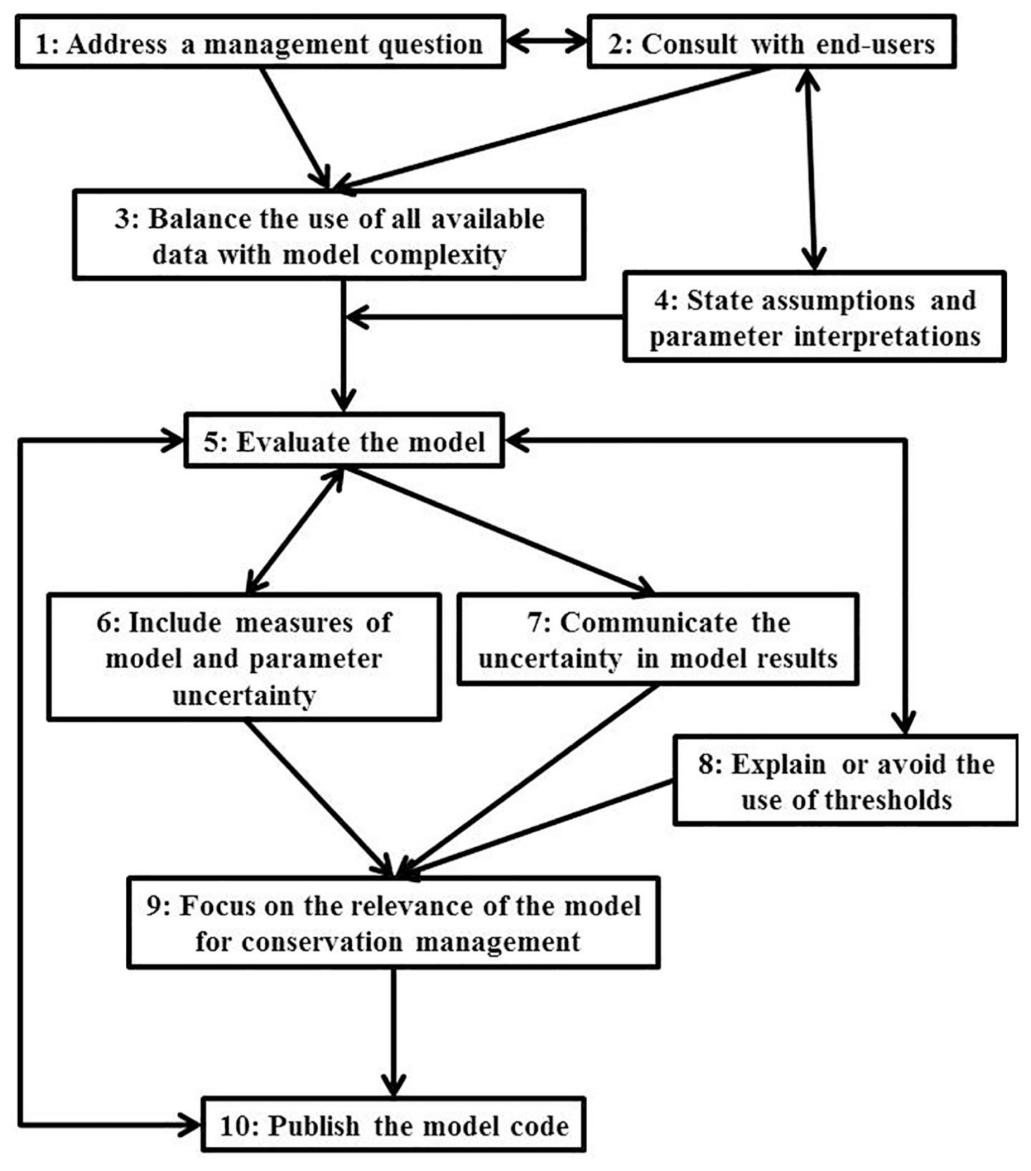
Ten recommendations, and their relationships, for best-practice in constructing quantitative models for conservation management. Arrows indicate the major connections between recommendations

The development of quantitative models to influence conservation management will benefit from guidelines that, on the one hand, can be used by modelers to construct fit-for-purpose models and, on the other, can be used by practitioners and end-users to benchmark the quality and reliability of any quantitative model (Addison et al., 2013; Conroy & Peterson, 2013; Guillera-Arroita, Lahoz-Monfort, Elith, et al., 2015; Schmolke et al., 2010). Drawing from our collective experience in the field of applied quantitative modeling to support and inform conservation decision-making, we present 10 general recommendations that can be applied to virtually any type of quantitative model used in conservation management (Figure 2). We have focused our recommendations on constructing and using applied models, once the data needed to populate these models have been acquired. Recent discussions on the role of good data for conservation management can be found elsewhere (Akçakaya et al., 2016; Joppa et al., 2016). It is not our intention to produce an exhaustive or a prescriptive list of recommendations, nor modeling approaches, and we acknowledge that there are multiple ways to develop models for informing conservation management (e.g., see Schmolke et al., 2010; Addison et al., 2013; Table 1). Instead, we propose that our 10 recommendations represent a minimum set of standards for constructing, using, and assessing conservation modeling. We illustrate our 10 recommendations with succinct examples taken from our own research and the scientific literature with which we are best familiar, that is, with a particular emphasis on Australian and New Zealand work given our scientific research background. Nonetheless, all of the examples provide lessons of broad relevance in the context of conservation management.

Our 10 recommendations are not necessarily independent (Figure 2). However, discussing them separately results in a clearer picture of their application and helps to comprehend where they fit within existing decision-making conventions and within ecological science (Addison et al., 2013; Akçakaya et al., 2016; Groves & Game, 2016; Schwartz et al., 2018). For clarity, and to facilitate their incorporation into modeling workflows, we have assigned each of the 10 recommendations to four stages of model construction: (a) design (two recommendations); (b) specification (two); (c) evaluation (one); and, (d) inference (five).

### 4.1 Model design

1. *Conceptualizing and developing a model to primarily address a conservation problem, not an ecological question, will produce a more valuable and longer-lasting resource for management*. The model will be most impactful if it is framed to address a real-world conservation problem. Answers to conservation questions are more likely to result in actions, such as the optimal strategy to allocate resources to achieve conservation objectives (Carwardine et al., 2012; Conroy & Peterson, 2013; McCarthy et al., 2010; Schmolke et al., 2010). This will also help foster a meaningful conversation and engagement with end-users (see next recommendation). Models addressing conservation issues commonly include ecological aspects, but it is not a pre-requisite. For example, some models to predict the unintentional transport of invasive species as stowaways in aeroplanes and ships do not include any ecological function, only estimates of transport pressure (e.g., see the transport model for alien amphibians in García-Díaz et al., 2017). Another good example is the different emphasis placed on the interest in detection versus occupancy in ecological versus conservation applications. In ecological research, imperfect detectability is usually treated as a nuisance parameter that contributes to false absences recorded for the target species (Kéry & Royle, 2016; Lahoz-Monfort, Guillera-Arroita, & Wintle, 2014). The converse is true of threatened species surveys and invasive species management, where the probability of detection is often the focal parameter of interest to guide surveillance efforts (Anderson et al., 2013; Garrard, Bekessy, McCarthy, & Wintle, 2015; Guillera-Arroita, Lahoz-Monfort, McCarthy, & Wintle, 2015).
  Nevertheless, it is still important to recognize that ecological models frequently underlie applied conservation models (Lurgi et al., 2015). For example, a population ecology model of invasive stoats (*Mustela erminea*) on Resolution Island, New Zealand was used to inform cost-effective management options to suppress their population density (Anderson, McMurtrie, Edge, Baxter, & Byrom, 2016).
2. *Consulting with end-users helps construct a sensible model*. Parrott (2017) recently proposed a framework for the collaborative construction of quantitative models in conservation management, and we refer readers to that publication for a detailed discussion of this topic. We observe that consultation and collaboration in developing a model do not need to rely on co-development (Addison et al., 2013; Wood, Stillman, & Goss-Custard, 2015). Conceptualizing and explaining the model and seeking feedback can often suffice, as end-users will not always be familiar with (or want to develop skills in) the specific modeling techniques. Modelers, however, will always benefit from end-users' knowledge of the system, and stakeholders who are consulted throughout the model development phase are more likely to adopt the conclusions drawn from modeling for conservation management (Addison et al., 2013; Parrott, 2017; Schmolke et al., 2010; Wood et al., 2015).

### 4.2 Model specification

3. *Balancing the use of all the relevant available data with model complexity supports conservation management in a* “*wicked world*”. Given that natural and social systems are complex and variable, our knowledge of them is affected by considerable uncertainty (Evans, Davila, Toomey, & Wyborn, 2017; Milner-Gulland, Shea, & Punt, 2017). It is therefore helpful to incorporate as much pertinent information as possible in the model. This will increase the likelihood that: (a) the model is representative of the existing knowledge; (b) knowledge gaps are identified; and, (c) unforeseen relationships are accounted for properly. However, this does not mean throwing the “kitchen-sink” into the model to generate an overly complex model, which can be difficult to interpret and communicate to end-users (Cartwright et al., 2016; Evans et al., 2013). Rather, it refers to specifying a model that accommodates all of the information assumed to influence the modeled processes while remaining sufficiently simple to address its conservation management purpose efficiently (i.e., “parsimony”). For instance, an overly complex model could result in model over-fitting (e.g., in species distribution models; Radosavljevic & Anderson, 2014) and difficulties in assessing the influence of different sub-processes on the overall system dynamics (e.g., spatially-explicit individual-based simulation models; Prowse et al., 2016; DeAngelis & Yurek, 2017). Fortunately, there are methodological techniques available to reach a reasonable trade-off between model complexity and the use of existing data. Examples include statistical regularisation for regressions (including all the covariates while also guarding against over-fitting; Gelman, Carlin, Stern, & Rubin, 2013), information-theory based multi-model inference (Burnham & Anderson, 2002; Dormann et al., 2018), machine learning methods for global *sensitivity analysis* of complex simulation models (Prowse et al., 2016), and integral projection models utilising a variety of data sources to model sub-processes within a main matrix population model (Ellner, Childs, & Rees, 2016; Saunders et al., 2018). In addition, Bayesian methods are a logical and effective way of incorporating preexisting (“prior”) information (Gelman et al., 2013; Hobbs & Hooten, 2015).
4. *Being clear about the assumptions, units, and interpretation of the parameters in the model helps avert unintended model-based conservation outcomes*. Lack of clarity about the units and meaning of the model parameters can lead to ambiguity or unintended consequences, and can potentially hinder acceptance by end-users (Cairney, Oliver, & Wellstead, 2016; Cartwright et al., 2016; Conroy & Peterson, 2013). A good example is the common misinterpretation of the complement of the probability of detection (1-P_detection_) as the probability of a species' absence given that it is not detected. The proper specification uses Bayes' rule and incorporates both the probability of not being detected and the probability of absence (Anderson et al., 2013; Guillera-Arroita, Lahoz-Monfort, McCarthy, & Wintle, 2015). Consulting with end-users during the construction of the model (recommendation 1) could reduce the likelihood of making untenable assumptions, and thus increases the likelihood of producing quantitative models that can genuinely influence conservation management.

### 4.3 Model evaluation

5. *Assessing the validity and adequacy of the model creates confidence in its reliability*. Model evaluation and *validation* against adequate suitability indicators, such as the percentage of variance and deviance explained (*R^2^* and *D^2^*, respectively) or the area under the receiver operative curve (however see Lobo, Jiménez-Valverde, & Real, 2008), will likely improve the confidence in its appropriateness to inform conservation management. In the case of a statistical model, the minimum requirement is an estimate of the goodness of fit of the model. It is important to keep in mind that *P*-values and information criteria scores such as Akaike's Information Criterion are not measures of model fit (Mac Nally, Duncan, Thomson, & Yen, 2018; Wasserstein & Lazar, 2016). When the intention is to use the model for prediction, projection, or extrapolation, the aim should be to validate the model with independent data or via cross-validation (Hobbs & Hooten, 2015; Hooten & Hobbs, 2015; Roberts et al., 2017; Rykiel, 1996; Sequeira, Bouchet, Yates, Mengersen, & Caley, 2018). In the case of simulation models, validation may not always be possible, but global sensitivity analyses can provide information on whether model outputs are robust to uncertainty in parameter inputs (Dietze, 2017; Getz et al., 2018; Prowse et al., 2016; Saltelli et al., 2008).

### 4.4 Model inference and use

**Figure 3:**
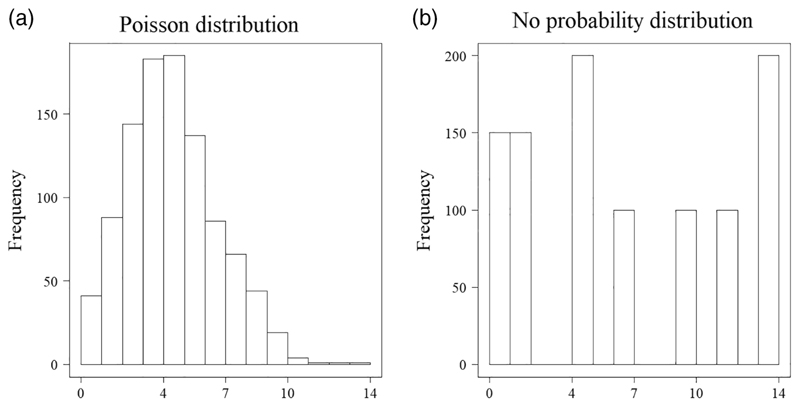
Two histograms illustrating recommendation 7 (“communicating the uncertainty in model results to end-users broadens its utility”). Both histograms represent distributions of values spanning the same range of values (x-axis), but only the one on the left follows a probability distribution (a Poisson distribution in this case). The histograms were obtained by plotting 1,000 random values drawn from a Poisson distribution with a mean of five (left panel) and 1,000 hand-picked values (right panel). R script to produce these graphs available from: https://gist.github.com/pablogarciadiaz/0ea50ffd31bb33263572dcfbcd3658ff

6. *Including measures of uncertainty when presenting inferences on model structure and model parameters is fundamental to informed conservation actions*. Uncertainty in model inferences is influenced by two main factors, which will contribute to the overall uncertainty and ambiguity in conservation actions (Chatfield, 2006; Dietze, 2017; Dietze et al., 2018; Milner-Gulland et al., 2017; Regan, Colyvan, & Burgman, 2002). First, the characteristics of the input data, including data sparseness in statistical models and the input data quality in simulation models, typically propagate through the model and produce uncertain parameter estimates. Second, the specification of the modeled processes leads to overall model uncertainty (also called structural uncertainty), indicating how close the current model is to be an accurate portrayal of the reality (Chatfield, 2006; Dietze et al., 2018). Model and parameter uncertainty measures complement other measures of centrality (e.g., mean or median). In the case of statistical models, familiar measures of parameter uncertainty are the standard deviation, standard error, and 95% confidence intervals (or credible intervals in the Bayesian framework). Model selection, multi-model inference, and model averaging using information criteria (e.g., Akaike's Information Criterion) and Bayesian posterior model probabilities, that is, the probability that a given model in a set of candidates is the best supported one, can contend with model uncertainty (Burnham & Anderson, 2002; Dormann et al., 2018; Hobbs & Hooten, 2015; Hooten & Hobbs, 2015; Kéry & Royle, 2016). Quantifying model and parameter uncertainty in simulation models is difficult due to the strong dependency of model specification and outcomes on the input estimates. In this case, sensitivity analyses can quantify uncertainty by estimating the effect of changes in input parameter values on the model outcomes (Dietze, 2017; Prowse et al., 2016; Saltelli et al., 2008). Furthermore, presenting the results of simulation models as a set of scenarios representing alternative uncertain species and system conditions is a good way to be explicit about uncertainty in conservation management (Akçakaya et al., 2016; Groves & Game, 2016; Mahmoud et al., 2009; Nicholson et al., 2018; Peterson, Cumming, & Carpenter, 2003).
7. *Communicating the uncertainty in model results to endusers broadens a model's utility*. The end-users of quantitative models tend to focus on the model outputs that will be the target of the conservation action, such as predictions of the probability of the presence of a threatened species (Addison et al., 2013; Garrard et al., 2015; Guillera-Arroita, Lahoz-Monfort, McCarthy, & Wintle, 2015). All model outputs have some degree of uncertainty. Therefore, further to providing measures of model and parameter uncertainty (recommendation 6), we advise reporting uncertainties in all of the model outputs and results. Distributions of the values of relevant quantities resulting from the model outputs provide a natural framework to handle and communicate uncertainties in modeling results. Roughly, a distribution of values can be conceptualized as anything that can be plotted as a histogram—it can follow a probability distribution but it is not a precondition (Figure 3). The collection of final population sizes obtained from running simulations of a population viability analysis is an example of an output distribution of values (Beissinger & McCullogh, 2002; McCarthy et al., 2003).
  There are a number of important advantages to implementing this recommendation. For example, output distributions can be readily manipulated in existing mathematical and statistical software, so further postmodeling processing can be undertaken. Propagating the uncertainty in parameters estimated from a statistical model that will be used in a simulation model is seamless when the outputs of the statistical model are distributions of values (Wade, 2002). Output distributions can be interpreted in terms of risk assessments, a key tool in conservation management, as distributions provide a measure of the likelihood of occurrence of an event (Burgman, 2005). Moreover, distributions are a core component of conservation decision-making techniques such as cost-effectiveness analyses and stochastic dominance (Canessa, Ewen, West, McCarthy, & Walshe, 2016; Carwardine et al., 2012; Groves & Game, 2016). The main shortcoming of this recommendation is that output distributions can be difficult to communicate to conservation managers (Cartwright et al., 2016; Hoffrage, Lindsey, Hertwig, & Gigerenzer, 2000; Parrott, 2017). Nonetheless, that is a hurdle that can be overcome through effective communication and translation, and we posit that the benefits of this recommendation outweigh the potential complications (Burgman, 2005; Cartwright et al., 2016; Dietze et al., 2018; Groves & Game, 2016; Parrott, 2017).
8. *Being explicit when using thresholds is crucial to providing transparent applications of model results*. There is a frequent desire for applying thresholds to model outputs, for example, by calculating *P*-values to estimate significance or transforming probabilities of occurrence into binary categories (predicted presence or absence). Thresholds can sometimes be arbitrary and misleading when they are used in the context of conservation management, and it always is important to explain and justify their use (Bestelmeyer, 2006; Field, Tyre, Jonzén, Rhodes, & Possingham, 2004; Liu, Berry, Dawson, & Pearson, 2005; Wasserstein & Lazar, 2016)
  The development of optimal thresholds to discontinue surveillance or the removal of invasive species, by estimating the costs and benefits associated with deploying different surveying efforts, is a good example of a well-designed and justifiable threshold in the context of conservation management (Gormley, Anderson, & Nugent, 2017; Regan, McCarthy, Baxter, Panetta, & Possingham, 2006; Rout, Kirkwood, Sutherland, Murphy, & McCarthy, 2014). Ecological tipping points, system thresholds that once exceeded can irreversibly shift the dynamics of the system, are important in conservation management (Groffman et al., 2006; Guntenspergen, 2014). These ecological tipping points can be identified using statistical models, such as piecewise regressions, and represent another prominent example of adequate statistical thresholds of relevance for conservation management (Ficetola & Denoël, 2009). Being explicit about thresholds when presenting model results guarantees transparency when interpreting, evaluating, and translating findings.
9. *It is important to recognize that a model evolves iteratively, and should not be the focus for conservation action*. The model, no matter how novel and interesting, is a means to help in achieving the goal of informing conservation management. The situation is slightly different when the model is part of an adaptive conservation management program (Addison et al., 2013; Conroy & Peterson, 2013; Dietze et al., 2018; Salafsky et al., 2016; Schmolke et al., 2010). In that case, the continuous updating and improvement of the model can become central to conservation management. As such, it is crucial to describe, justify, and evaluate its appropriateness. Quantitative models used to guide marine fisheries quotas are regularly revised to reflect the evolving status of such fisheries (e.g., see the International Commission for the Conservation of Atlantic Tunas: https://www.iccat.int/en/assess.htm). However, in all cases, the focus of the research should be on the results and outputs of the model, and how they are relevant for conservation management. It remains appropriate to always be considerate of ways to improve the models as required.
10. *Annotating the model code and making it available publicly fosters reproducibility and repeatability*. Being properly annotated and publicly accessible, the model becomes reproducible and subject to scrutiny that can enhance its quality and assist in verifying its validity. This will also allow for the model's timely revision and update when new information becomes available to both researchers and end-users (Barnes, 2010; LeVeque, 2013). There are multiple online platforms providing storage and facilitating version control for model code, including the popular repositories GitHub (https://github.com/) and Code Share (https://codeshare.io/). Sharing of code and programs should be a goal whenever possible.

## 5 Conclusions

Quantitative models have served an important role in generating effective conservation actions (Addison et al., 2013; Brook et al., 2000; Conroy & Peterson, 2013; McCarthy et al., 2010; Nicholson et al., 2018; Schmolke et al., 2010). In particular, we echo the recent call made by the IPBES for using models in biodiversity conservation (Akçakaya et al., 2016). This can be achieved if quantitative modelers conceptualize their models with the ultimate aim of informing conservation management (recommendation 1) and communicate with potential stakeholders (recommendation 2) from the outset of the research project (Addison et al., 2013; Parrott, 2017; Wood et al., 2015). Otherwise, building quantitative models, and subsequently attempting to apply them to management, can risk limiting the adoption of the results by the conservation management community.

Regardless, conserving biodiversity is a pressing and difficult task and one that we believe (along with many others) will benefit from reliable and robust quantitative models in its quest for success (Addison et al., 2013; Conroy & Peterson, 2013; McCarthy et al., 2004; Nicholson et al., 2018; Schmolke et al., 2010). In this review, we have described the role of effective models in conservation management and outlined ten general recommendations for the development, use, application, and translation of, sound models. We are confident that the thorough application of our recommendations will increase the impact of quantitative models on conservation outcomes. Finally, quantitative modeling is a diverse field with multiple perspectives, and we hope that our review will contribute to the discussion on the use and misuse of quantitative models in conservation management.

## Figures and Tables

**Table 1:** A comparison of the 10 quantitative modeling recommendations proposed in this review, and their occurrence in two previous reviews of quantitative modeling in conservation management

Recommendation/publication	This review	Schmolke et al. (2010)^a^	Addison et al. (2013)^b^
Address a management question	✓		✓
Consult with end-users	✓		✓
Balance the use of all available data with model complexity	✓		
State assumptions and parameter interpretations	✓	✓	
Evaluate the model	✓	✓	
Include measures of model and parameter uncertainty	✓	✓	
Communicate the uncertainty in model results	✓	✓	✓
Explain or avoid the use of thresholds	✓		
Focus on the relevance of the model for conservation management	✓	✓	✓
Publish the model code	✓		

Note that we focus on explicit occurrences of the recommendations, whereas other broader recommendations (e.g., defining the context and audience of the model; from Box 1 in Schmolke et al., 2010) are not included. Moreover, the terminology differs across the three reviews and this table is subsequently subject to some degree of interpretation.

a Assessed from Box 1 in Schmolke et al. (2010).

b Assessed from Table 2 in Addison et al. (2013).

## Glossary

*Conservation management*: activities conducted with the primary aim of conserving species and systems to achieve maintenance or restoration of biodiversity features.

*Correlative or correlational model*: a model representing noncausative associations between two or more variables.

*Differential equation*: Mathematical equation that relates a function with one or more of its derivatives (i.e., the instantaneous rate of change of a function). For example, an expression for the rate of change (in space or time) of a population.

*Estimation*: using data to approximate, with some degree of uncertainty, the parameter values of a model.

*Gene drive*: a synthetic, self-replicating genetic element that can propagate one or more focal genes through a population.

*Individual-based model*: a model where individuals are the basic units at which the parameterization and estimation occur.

*Mechanistic model*: a model explicitly defining the process being modeled (also termed “process model”).

*Model parameters*: broadly defined, parameters are any component of a model that can be measured or estimated (e.g., the slopes in a statistical model or the population growth rate in a population model).

*Model validation*: using independent data to assess whether an existing model produces predictions consistent with repeated observations and real-world processes.

*Prediction*: using a model to infer, with some degree of uncertainty, the trajectories of a system or process of interest under a set of conditions different to those used to construct the model (e.g., projection into the future or to a different geographical area). It is commonly considered synonymous with forecasting.

*Population viability analysis*: techniques used to project the likelihood of a population, of a given abundance, surviving for a given number of years into the future.

*Quantitative model*: a model that endeavors to describe and/or forecast the behavior of a system using mathematical and statistical concepts, and whose parameters and their relationships are expressed as quantities.

*Sensitivity analysis*: an analytical method to assess how a change in the value of a parameter in a model influences the value of another parameter(s) in the model.

*Species distribution model*: a model constructed to explain and predict the occurrence of a species.

*Simulation model*: a model constructed to replicate the system, and populated (also sometimes called parameterisation) with parameter estimates usually borrowed from separate data sources and independent statistical models.

*Statistical model*: broadly defined, those models fitted (also called sometimes calibrated or parameterized) to existing data using statistical methods, with the underlying structure ranging from a mechanistic to a correlative model.

*Strategic model*: a simplified mechanistic model representing causal relationships between parameters.

*“Wicked” conservation problem*: a conservation management problem characterized by being highly complex and lacking a single optimal solution. Most frequently, there are many high-order interactions between variables (e.g., environmental factors) and actors with different goals and perspectives (e.g., scientists, the public, the government), producing substantial uncertainty and difficult decision trade-offs.
